# Impacted bone allograft personalised by a novel 3D printed customization kit produces high surgical accuracy in medial opening wedge high tibial osteotomy: a pilot study

**DOI:** 10.1186/s40634-023-00593-0

**Published:** 2023-03-14

**Authors:** Wouter Van Genechten, Annemieke Van Haver, Stijn Bartholomeeusen, Toon Claes, Nathalie Van Beek, Jozef Michielsen, Steven Claes, Peter Verdonk

**Affiliations:** 1 Orthopedic Department, University Hospital Antwerp, Antwerp, Belgium; 2 More Institute, Antwerp, Belgium; 3 Orthopedic Department, AZ Herentals, Herentals, Belgium; 4 ORTHOCA, Antwerp, Belgium

**Keywords:** High tibial osteotomy, 3D planning, Patient-specific instrumentation, Accuracy, Joint preservation

## Abstract

**Purpose:**

Contemporary medial opening wedge high tibial osteotomy (MOWHTO) still seems to struggle with inconsistent accuracy outcomes. Our objective was to assess surgical accuracy and short-term clinical outcomes when using 3D planning and a patient-specific instrumentation (PSI) kit to prepare customized bone allografts.

**Methods:**

Thirty subjects (age 48y ± 13) were included in a double-center prospective case series. A low-dose CT-scan was performed to generate 3D bone models, a MOWHTO was simulated, and PSI was designed and 3D printed based on the complementary negative of the planned osteotomy gap. Clinical outcome was assessed at two, four, 12 weeks and one year using NRS, KOOS, UCLA activity score, EQ-5D and anchor questions. A linear-mixed model approach was implemented for data analysis.

**Results:**

Preoperative 3D values were 175.0° ± 2.2 mechanical tibiofemoral angle (mTFA), 85.0° ± 3.0 medial proximal tibial angle (MPTA), and 94.1° ± 3.4 medial posterior tibial slope (MPTS). Target planning ranged from slight varus to the lateral tibial spine (slight valgus). Postoperative 3D analysis showed an accuracy of 1.1° ± 0.7 ΔMPTA (*p* = 0.04) and 1.2° ± 1.2 ΔMPTS (*p* = 0.11). NRS decreased from baseline 6.1 ± 1.9 to 2.7 ± 1.9 at four weeks (*p* < 0.001) and 1.7 ± 1.9 at one year (*p* < 0.001). KOOS increased from 31.4 ± 17.6 to 50.6 ± 20.6 at 12 weeks (*p* < 0.001) and to 71.8 ± 15.6 at one year (*p* < 0.001).

**Conclusion:**

The study suggests that 3D printed instrumentation to personalize structural bone allograft is a viable alternative method in MOWHTO that has the benefit of optimizing surgical accuracy while providing early and consistent pain relief after surgery.

## Introduction

Medial opening wedge high tibial osteotomy (MOWHTO) is an established procedure to correct varus malalignment of the lower limb, primarily indicated for isolated osteoarthritis and focal cartilage lesions in medial compartment of the knee or ligamentous instability [1]. Over the past decades, there has been a general decline in MOWHTO performance in Europe and North America, even though excellent survival rates are reported with favorable clinical and radiological outcomes in the young and active patient [2].

Contemporary MOWHTO still seems to struggle with inconsistent accuracy outcomes [1]. A systematic review from 2016 uncovered a fairly low surgical accuracy relative to the proposed planning by conventional MOWHTO techniques; eight out of 14 cohorts (57%) reported an accuracy rate below 75% within a self-defined accuracy interval [1]. The majority of inaccurate cases appeared to be under-corrected [1]. Reasons for low accuracy outcomes may lie in unprecise 2D planning of the osteotomy, the challenging translation of the planned correction, postoperative soft tissue rebalancing and loss of correction due to unstable hinge fractures [3–5]. Measuring errors might not be surprising, given that the majority of MOWHTO planning is solely based on a single full-leg bipedal standing radiograph (FLSR) [6].

Besides under-correction in the coronal plane, an unintended tibial slope increase in the sagittal plane often cannot be avoided after conventional MOWHTO [7, 8]. Although the actual slope change might be of minor clinical relevance in the majority of patients, Kim et al. found degenerative changes of the ACL on second-look arthroscopy in a subgroup of patients with higher BMI and an excessive tibial slope increase [9]. Ultimately, obtaining the planned correction in MOWHTO is considered a highly important factor as long-term clinical results depend on the accuracy of the lower limb realignment [10]. Recently, a bone allograft impaction technique revealed promising results on early pain scores, weight-bearing and initial construct stability after MOWHTO, which justifies further research on this topic [11, 12]. The study aims to assess surgical accuracy and short-term clinical outcomes when using 3D planning and a patient-specific instrumentation (PSI) kit to prepare customized impacted bone allografts.

## Methods

A two center prospective case series was conducted involving the orthopedic departments of AZ Monica, Belgium and AZ Herentals, Belgium. From September 2020 to October 2021, patients for whom an MOWHTO was indicated, were screened for study inclusion according to the following criteria: symptomatic isolated medial knee osteoarthritis evidenced by radiographs (Kellgren-Lawrence grade 1–4), varus alignment on full-leg standing radiograph (mechanical tibiofemoral angle (mTFA) < 178°) and age > 18 years. Concomitant procedures such as knee arthroscopy, anterior cruciate ligament (ACL)/anterolateral ligament (ALL) reconstruction and cartilage restoration were allowed per protocol. Exclusion criteria were extreme varus malalignment (mTFA < 165°), preoperative range of motion (ROM) < 100°, significant collateral ligament laxity, bilateral simultaneous HTO, any systemic inflammatory condition (e.g. rheumatic disorders, Sjörgens disease…) and any of the following medical disorders or factor: active psychiatric or neurologic diseases, active alcohol or drug abuse, unwilling to stop smoking for eight weeks.

Study was approved by the university and local ethical committees on 06/10/2020 (#B3002020000026). Written informed consent was obtained from all subjects preceding participation. The study was conducted in accordance with the Helsinki Declaration, the European Union Directive on Medical Devices (93/42 / EEC art.15), the guidelines related to clinical studies as outlined in EN ISO 14155 and in agreement with the rules of good clinical practice.

### Preoperative imaging, 3D planning and customized kit

A preoperative standardized FLSR (2D) was part of the pre-study diagnostic work-up and so available in every patient. After study enrollment, subjects received a low-dose CT-scan of the whole index limb according to the Trumatch protocol by Depuy-Synthes®. This protocol involves scanning of the hip and ankle joint on a 5 mm thickness and spacing, and the knee on a 0.5 mm thickness and spacing, captured in 150 mm centered range. Digital imaging and communications in medicine (DICOM)-files were loaded in medical image software Mimics 23.0 (Materialise®, Leuven, Belgium). Threshold or segmentation was set at 130–200 Hounsfield units (HU) and unrequired bone parts such as the acetabular socket and talus were manually removed. The final 3D reconstruction of the lower limb was exported as Stereolithography (STL)-files and opened in medical 3D planning software 3-matic 14.0 (Materialise®, Leuven, Belgium).

**Fig. 1:**
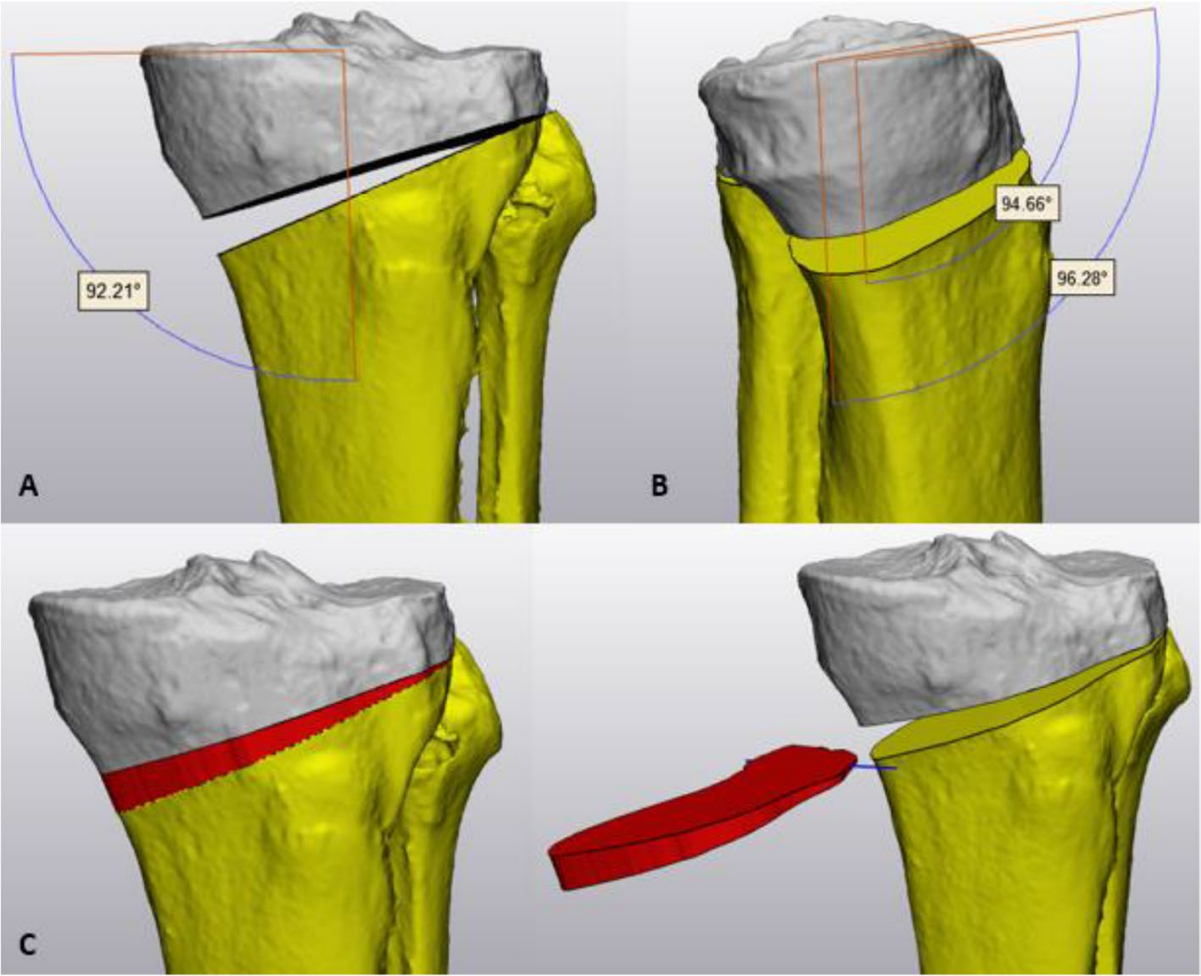
3D preoperative osteotomy simulation using (**A**) the planning angles MPTA in the coronal plane and (**B**) the tibial slopes in the sagittal plane. (**C**) The negative of the planned osteotomy gap (red) is embodied and exported with the hinge axis (blue) to design the 3D printed customization kit for bone graft preparation

**Fig. 2:**
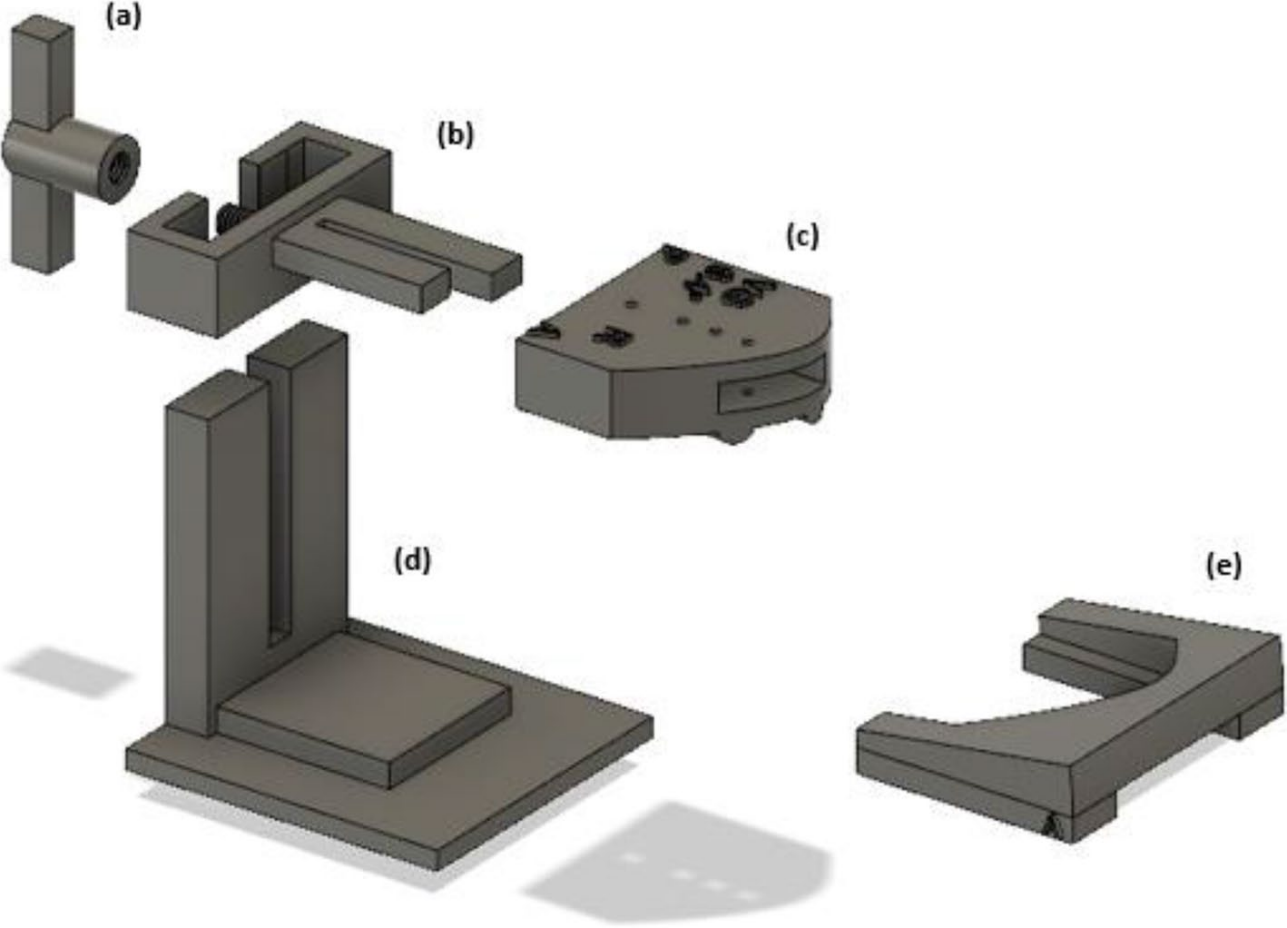
Design of the customized bone graft preparation kit: (**a**) a winged nut, (**b**) an adjustable upper fixation part, (**c**) a cutting block, (**d**) a backed platform and (**e**) a sliding cast of the required gap opening. Cutting block (c) is marked with patients’ initials, side of surgery, correction size and anteroposterior graft orientation

The hip center was determined by marking the femoral head with subsequent fitting of a best fit sphere. The center of the distal tibia (pilon) was defined by measuring the anteroposterior and mediolateral middle of the tibia plafond surface. Correct positioning was visually controlled on a anteroposterior view. Landmarks around the knee joint were defined as described by Victor et al. [13]. The anatomic plane of the femur was determined by connecting the medial and lateral epicondyle of the femur and the femoral head center. The mechanical femoral axis was created by connecting the femoral head center and the middle of the trans-epicondylar axis (TEA). The anatomic plane of the tibia was defined by the tip of the medial and lateral tibial spine and the pilon center. The mechanical tibial axis was created by connecting the center of the tibial plafond to the middle of the medial and lateral spine distance. The lateral distal femoral angle (LDFA), the medial proximal tibial angle (MPTA) and the mTFA were measured in the coronal plane while the medial and lateral posterior slope were determined in the sagittal plane. For osteotomy planning, an individualized target was preferred based on the pre-existing tibial varus (MPTA), overall varus degree (mTFA) and alignment of the contralateral side, but never exceeding a planned MPTA > 96°. The target angle ranged from slight varus towards crossing the weight-bearing line (WBL) through the lateral tibial spine (slight valgus). For osteotomy simulation, a cutting plane was designed starting from the vertical convex-concave transition of the medial proximal tibia, approximately 35 mm below the tibia plateau which was directed towards the tip of the fibular head. The plane offset was set at 0.9 mm, corresponding to the actual sawblade thickness and intraoperative bone loss. Next, the hinge axis was determined at 5-10 mm from the lateral cortex and perpendicular to the posterior tibial condylar line. The osteotomy was opened until the desired MPTA and tibial slope were obtained (Fig. 1A/B). Subsequently, the negative of the created osteotomy gap was embodied and together with the hinge axis exported for design of the customization bone graft preparation kit (Fig. 1C). Each kit consists of five parts: (a) a winged nut, (b) an adjustable upper fixation part, (c) a cutting block, (d) a backed platform and (e) a sliding cast of the required gap opening (Fig. 2). Cutting blocks (c) were marked with patients’ initials, side of surgery, correction size and anteroposterior graft orientation. The kit was 3D printed (OCEANZ®, Ede, Netherlands) in medical-grade biocompatible polyamide 12 (ISO-13485) and sterilized in an autoclave with saturated steam at 134 °C for three and a half hours.

### Surgical technique

**Fig. 3:**
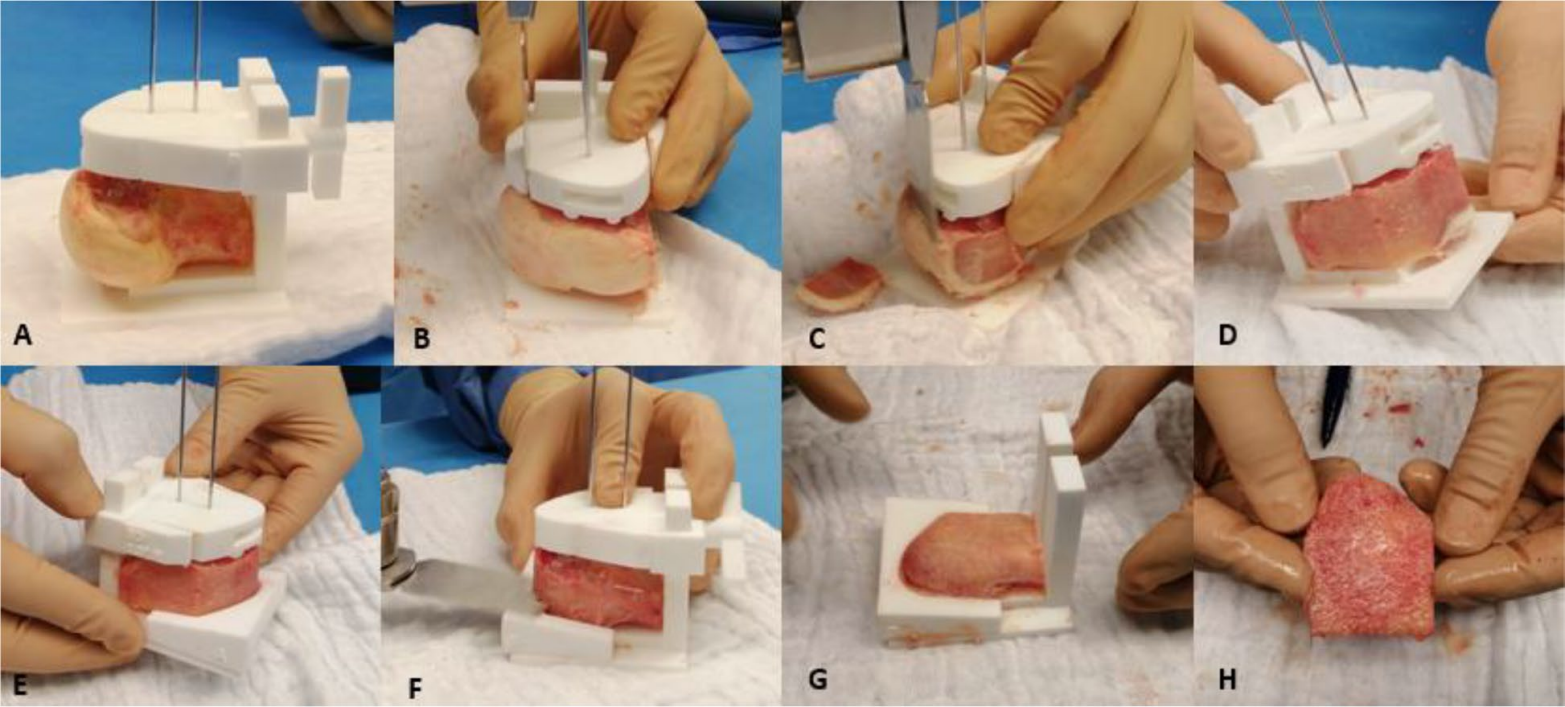
Intraoperative preparation of structural impacted bone allograft with the customized 3D printed kit. (**A**) The femoral head is placed on the platform and fixed with two 1.8 mm Kirschner pins through the cutting block, without full engagement to the bottom. (**B**) The anterior and posterior borders of the graft are cut perpendicular to the platform. (**C**) The medial contour of the graft is trimmed, which identically matches the curvature of the medial cortex of the patient. Correct graft positioning can hereby later controlled. (**D**) The desired result after graft shape contouring with an oscillating saw. (**E**) Next, the designated cast is pushed to the graft, again matching the prepared medial curvature of the graft. (**F**) The upper surface of the cast is used as guiding plane to obtain (**G**) the desired bone wedge. (**H**) Finally, the intended structural bone allograft can safely be removed from the guide and is ready for introduction in the osteotomy gap

Four senior knee surgeons contributed to the study. A conventional uniplanar (33%) or biplanar (66%) medial opening wedge HTO procedure was performed as previously described [12, 14]. As preplanned, the osteotomy was started at the vertical convex-concave transition of the medial proximal tibia, approximately 35 mm below the tibia plateau and was directed towards the tip of the fibular head. The superficial medial collateral ligament was first released while the pes anserinus remained untouched. The osteotomy was then performed and gradually opened by inserting multiple chisels (DepuySynthes®) or by use of a screwed spreader device (Arthrex®). While allowing the lateral cortical hinge to accommodate in this position, attention was directed to the customized bone graft preparation kit (Fig. 3). A fresh-frozen femoral head from the tissue bank was used to manufacture the customized impacted bone graft. The allograft was fixed with two 1.8 mm Kirschner pins through the cutting block. The anterior and posterior edges of the graft were first cut perpendicular to the platform followed by the medial cortical contour of the graft. Next all debris was discarded and the designated cast was shifted over the platform encasing the graft at the anterior, posterior and medial side. The upper surface of the cast was used as guiding plane to make the final horizontal cut at the proximal side of the graft in order to obtain the desired bone wedge dimensions. The customized bone graft was then introduced using a horseshoe-like instrument (Gaplocker®) or lamina spreader that maintained the osteotomy opening. Attention was paid for matching the medial contours of both tibial cortex and allograft which ultimately indicated proper press-fit graft orientation. Correct graft positioning was checked under fluoroscopy. A locking plate (depending on the surgeons preference) was finally applied to stabilize the osteotomy construct.

Postoperative weight-bearing was allowed as tolerated (but not mandatory) from the first day. All subjects had a physiotherapy session on the orthopaedic ward before discharge the day after surgery. Acetaminophen (PO, max four g/day) and tramadol 50 mg (PO, max 200 mg/day) were prescribed for ambulatory pain control. After four weeks, physiotherapy sessions were initiated if deemed required by the treating physician.

### Patient-reported outcome measures (PROMs)

Short-term clinical outcomes were assessed by the Numeric rating scale (NRS) for pain, the knee injury and osteoarthritis outcome score (KOOS), the UCLA activity score and the EQ-5D global assessment score at baseline and two, four, 12 weeks and one year after surgery. At four weeks, specific anchor questions were asked about the use of walking aids and the ability of car driving.

### Postoperative imaging and accuracy

**Fig. 4:**
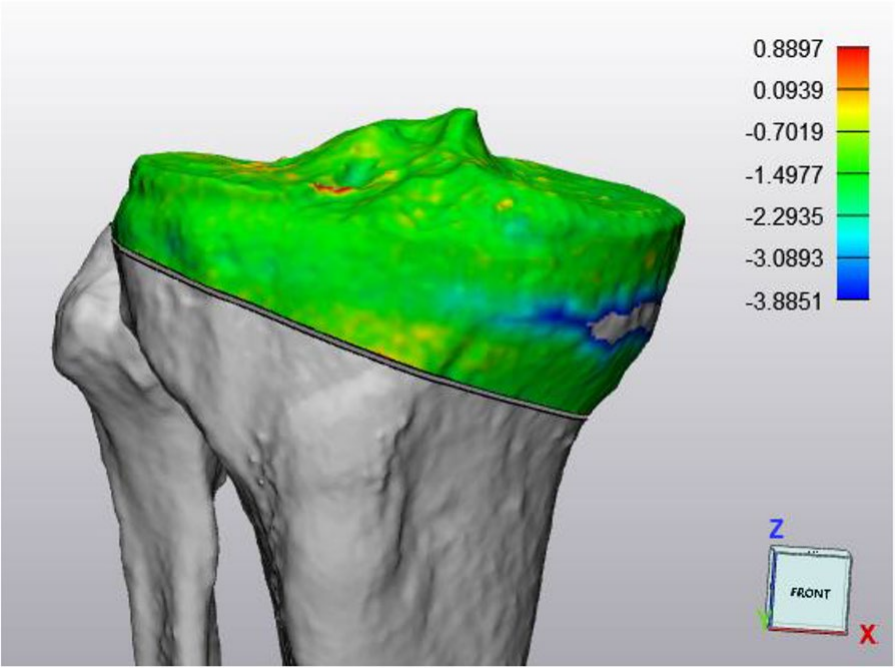
3D matching of the pre-and postoperative proximal tibia plateaus, above the osteotomy level. Average distance error between bone models was 0.0716 ± 0.0019 mm

**Fig. 5:**
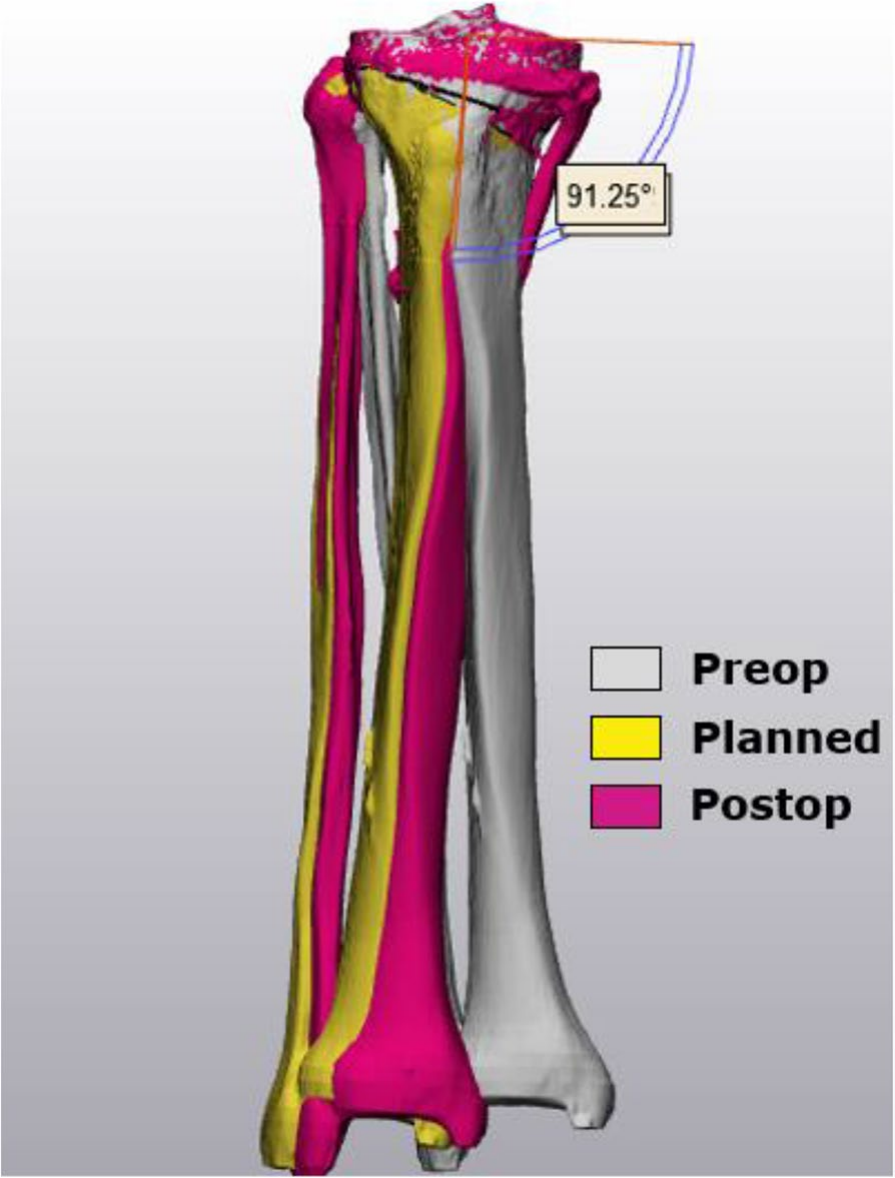
Postoperative 3D accuracy analysis by matching the pre-and postoperative proximal tibia plateaus

A CT-scan of the knee and ankle, with the same parameters as the preoperative CT-scan, was repeated at three months postoperative to assess biplanar accuracy, bone union and lateral hinge fractures [15]. Standard knee radiographs and a full-leg standing radiograph were also provided at three months and knee radiographs repeated at one year after surgery. Equal thresholds were applied during 3D bone segmentation (130–200 HU). To allow precise accuracy measurements, the proximal tibia plateau (above the osteotomy) was matched with the preoperative ‘planned’ model using the global registration-tool (Fig. 4). Only the pilon point had to be redefined on the postoperative 3D model. All other preoperative planning landmarks, axes and planes were re-used to rule out measurement errors (Fig. 5). The preferred accuracy method was the ΔMPTA (postoperative – planned MPTA) in the coronal plane and the medial posterior tibial slope (ΔMPTS; postoperative – planned MPTS) in the sagittal plane. Accuracy outcomes were expressed both as relative (x) and absolute values (|x|).

### Statistics

Descriptive statistical analysis were conducted to provide an overview of the patients’ characteristics and radiological measurements in mean, standard deviation (SD), and minimum/maximum range []. First, normal distribution was assessed by Shapiro–Wilk test without exclusion of outliers. Outcomes for normal distribution guided further statistics into parametric or non-parametric tests. In case where data were normally distributed, paired Student’s t-test were performed to compare planned with postoperative angles. Based on this analysis, surgical accuracy in both the coronal and the sagittal plane could be determined. When data was not normally distributed, the non-parametric Mann–Whitney test was used. For each clinical parameter of interest, a linear-mixed model repeated measures approach (LMM) was implemented. Alpha was set at 0.05 to define statistical significance. Statistical tests were conducted in Graphpad 8.0. (IBM Co., Armonk, NY, USA) and R Core Team (2013. Vienna, Austria: R Foundation for Statistical Computing).

## Results

Thirty subjects (31 surgeries) were enrolled and received an MOWHTO with customized impacted bone allograft. The indication for MOWHTO was isolated medial OA (90%), focal medal cartilage defect (3%) and ligamentous instability (6%). One subject had a consecutive bilateral HTO within three and a half months, with both surgeries included in the study. A single subject was excluded for analysis because of immediate correction loss due to plate mal-positioning (surgical error). Study demographics and preoperative angles of the remaining 30 analyzed HTO surgeries are outlined in Table 1. Six subjects (20%) had a concomitant index knee procedure in the form of a knee arthroscopy (*n* = 3, 11%), implantation of a metal resurfacing button on the medial femoral condyle (*n* = 1, 3%), an ACL reconstruction (*n* = 1, 3%) or a mono-loop ALL reconstruction (*n* = 1, 3%).

**Table 1:** Baseline patient characteristics. Severity of OA was scored according to the Kellgren- Lawrence classification. Preoperative angles were measured on full leg standing radiographs (2D) or in 3D software on non-weightbearing CT-scan (BMI, body mass index; OA, osteoarthritis; MPTA, medial proximal tibial angle mTFA, mechanical tibiofemoral angle; LDFA, lateral distal femoral angle; MPTS, medial posterior tibial slope)

Parameter	Analysed group (*n* = 30)
**Age (years), mean ± SD [range]**	48 ± 13 [18 to 70]
**Male, n (%)**	27 (90)
**Right, n (%)**	16 (53)
**BMI, mean ± SD [range]**	27.9 ± 5.1 [16.9–37.0]
**MPTA (°), mean ± SD [range]**	3D| 85.0° ± 3.0 [76.6–88.9]
	2D| 85.2° ± 2.5 [78.0–89.3]
**mTFA (°), mean ± SD [range]**	3D| 175.0° ± 2.2 [169.3–177.9]
	2D| 174.2° ± 2.4 [167.5–177.2]
**LDFA (°), mean ± SD [range]**	3D| 87.6° ± 1.4 [84.8–90.7]
	2D| 88.2° ± 1.6 [85.1–92.7]
**MPTS (°), mean ± SD [range]**	3D| 94.1° ± 3.4 [87.9–102.3]
**Severity OA, n (%)**	
** Grade 1**	8 (27%)
** Grade 2**	8 (27%)
** Grade 3**	11 (36%)
** Grade 4**	3 (10%)

### Accuracy outcomes, complications and bone healing

The planned MPTA was 91.9° ± 2.6 [84.1–95.7] and mTFA was 181.9° ± 2.0 [178.3–184.8]. The average planned correction size was 6.9° ± 1.1 [4.8–9.4]. In the sagittal plane, no major corrections were desired, leaving the MPTS at 94.1° ± 3.0 [88.6–102.4] after planning. By matching the preoperative with the postoperative proximal tibia for accuracy analysis, the average distance error between bone models was 0.0716 ± 0.0019 mm. 3D accuracy outcomes (Table 2) in the coronal plane were -0.8° ± 1 [-3.0 to 1.9] relative ΔMPTA and 1.1° ± 0.7 [0.1–3.0°] absolute ΔMPTA (*p* = 0.04). The absolute MPTS deviation was 1.2° ± 1.2 [0.1–5.1°] (n.s.). In 63%, the obtained correction (MPTA) was falling within 1° around the planned target, while 90% fell into the < 2° range. All osteotomies fell within < 3° around the target. In the sagittal plane, the MPTS did not alter more than 2° in 87%. Five (16%) lateral hinge fractures were observed on postoperative CT-scan (three type I, one type II and one type III Takeuchi), while none were noticeable on conventional fluoroscopy. Fractures were undisplaced without the need for additional fixation. Beginning to advanced bone graft incorporation was observed three months after surgery on CT-scan while all osteotomies were consolidated at one year on plain radiographs. One minor postoperative bleeding the day after surgery was observed which was conservatively managed by compression therapy. One patient presented with a deep infection distally at the plate two months after surgery which was treated with open debridement and both local and IV antibiotics. Knee radiograph at six months showed progressive consolidation which was completed at one year. Another subject had a delayed union (no consolidation at six months) of unknown origin which was conservatively managed. CT-scan at one year revealed complete consolidation. Previous MOWHTO surgery on the contralateral side however showed a similar delayed healing pattern. Finally, five patients had their implant removed within the first year (7.8 months ± 3.6) for local irritation.

**Table 2:** The planned, postoperative and accuracy outcomes in 3D and 2D. (MPTA, medial proximal tibial angle mTFA, mechanical tibiofemoral angle; MPTS, medial posterior tibial slope)

**Angle**	Planned	Postoperative	*P*-value	Relative accuracy (x)	Absolute accuracy (|x|)
**MPTA (°), mean ± SD [range]**	3D| 91.9° ± 2.6 [84.1–95.7]	3D| 91.1° ± 2.3 [85.1–95.9]	0.04	-0.8° ± 1.0 [-3.0 to 1.9]	1.1° ± 0.7 [0.1–3.0°]
	2D| -	2D| 91.5° ± 1.7 [86.6–95.13]	-	-	-
**mTFA (°), mean ± SD [range]**	3D| 181.9° ± 2.0 [178.3–184.8]	3D| 181.1° ± 1.8 [176.1–183.2]	n.s	-0.8° ± 1.0 [-3.0 to 1.9]	1.0° ± 0.8 [0.1–3.0°]
	2D| -	2D| 181.0° ± 1.8 [176.6–183.3]	-	-	-
**MPTS (°), mean ± SD [range]**	3D| 94.1° ± 3.0 [88.6–102.4]	3D| 94.6° ± 3.6 [88.2–104.3]	n.s	0.5° ± 1.6 [-3.2–5.1°]	1.2° ± 1.2 [0.1–5.1°]

### Clinical outcomes

**Fig. 6:**
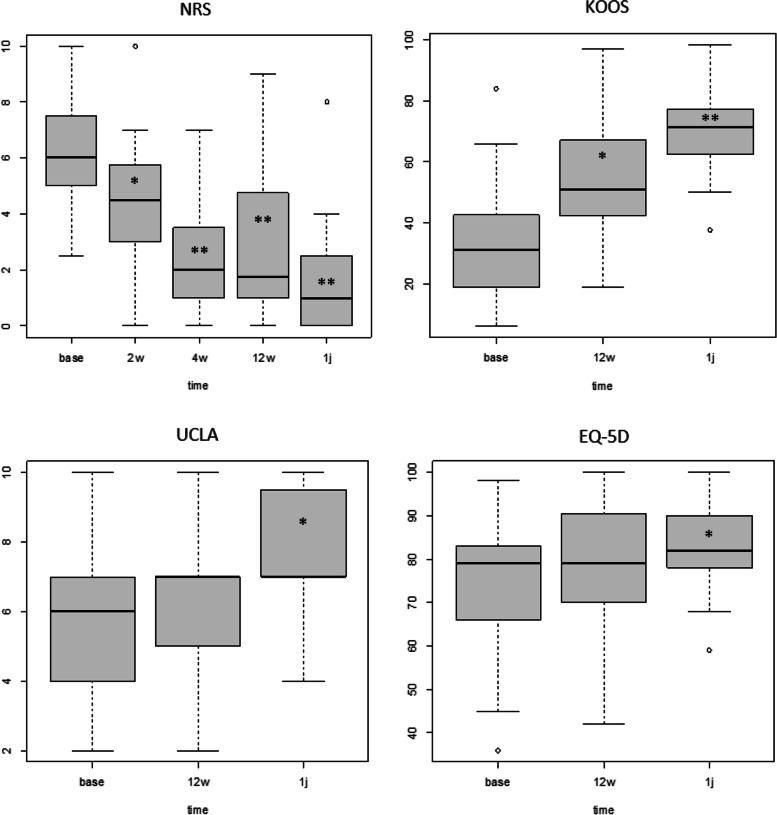
The Numeric Rating Scale (NRS), KOOS, UCLA and EQ-5D outcomes up to one year after surgery. (*significant difference compared to baseline; **significant difference compared to baseline and first postoperative timepoint)

The NRS pain score decreased from 6.1 ± 1.9 at baseline to 4.5 ± 2.1 at two weeks (*p* = 0.010), to 2.7 ± 1.9 at four weeks (*p* < 0.001) and to 2.9 ± 2.3 at 12 weeks (*p* < 0.001) after surgery (Fig. 6). After four weeks up to one year postoperatively (NRS 1.7 ± 1.9), no significant decrease in NRS was observed. KOOS outcome was 31.4 ± 17.6 preoperatively and increased to 50.6 ± 20.6 at 12 weeks (*p* < 0.001) and to 70.2 ± 15.0 at one year (*p* < 0.001) (Fig. 6). Baseline UCLA activity score was 5.7 ± 2.3, which increased to 6.1 ± 1.9 at 12 weeks (n.s) and to 7.6 ± 2.2 at one year (*p* = 0.002) (Fig. 6). The preoperative EQ-5D score was 71.8 ± 15.6 and increased to 76.6 ± 15.1 at 12 weeks (n.s.) and to 83.2 ± 11.4 at one year after surgery (*p* = 0.008) (Fig. 6). Anchor questions at four weeks revealed that 60% was able to drive a car and 80% was able to walk with one crutch or without any.

## Discussion

The main study findings are that by using the 3D printed customization kit for bone allograft preparation in MOWHTO, accuracy outcomes are 1.1° ± 0.7 absolute ΔMPTA (*p* = 0.04) with 63% of cases falling within [-1°; + 1°] and 90% within [-2°; + 2°] around the target. In the sagittal plane, a minor tibial slope increase of 1.2° ± 1.2 ΔMPTS was found relative to the planning (n.s.). Pain levels rapidly decreased in the first four weeks after surgery while ‘weight-bearing as tolerated’ was allowed from the first postoperative day.

The concept of customizing structural bone allograft to enhance surgical accuracy in MOWHTO originates from a pilot study published in 2020 [16]. The preoperative 3D planning remained unchanged in the current study, however modifications to guide design have facilitated graft preparation intraoperatively with special attention to maximize filling of the osteotomy gap (antero-posterior) and to assure correct posterior slope. The latter is reflected by the absolute accuracy outcomes in the sagittal plane being 1.2° ± 1.2 compared to 2.7° ± 1.8 in the initial PSI study [16]. One case of the current study had an unintended slope increase of 5.1°. It was hypothesized that this bone graft was correctly prepared but conversely introduced (antero-posterior flip) by the surgeon.

Since the first publication on the use of PSI for knee osteotomies [17], several surgeons have focused on the implementation of CT-based 3D planning and personalized guides in order to advance accuracy outcomes [18–22]. The systematic review by Van den Bempt et al., uncovered a critical problem concerning accuracy outcomes with conventional MOWHTO techniques, mainly featured by undercorrected cases [1]. Computer navigation has long been considered a potential solution for inaccurate osteotomy outcomes [23], nevertheless, due to time-consuming setup of equipment, additional cost and long learning curve, it never became the gold standard for MOWHTO. Moreover, in a level-one randomized control trial (RCT), Schröter et al. found no difference in absolute accuracy between computer navigation and the gap measurement method [24]. Since no conventional control group was included in our study, the absolute accuracy outcome in our series (1.1° ± 0.7) appears to be at least numerically in favor compared to the navigation (2.1° ± 1.4) and gap measurement group (1.7° ± 1.2) as described in the RCT [24].

Regarding other PSI techniques, the 3D accuracy outcomes in both coronal and sagittal plane are comparable with the pilot study by Munier et al. [21]. They found 100% MPTA accuracy within 2° around the planning while showing 90% accuracy in the sagittal plane (MPTS) [21]. This PSI technique was later investigated on a large population and showed relative accuracy outcomes of 0.5° ± 0.6 ΔMPTA and 1.0° ± 0.9 ΔmTFA [19]. Although the ΔMPTA was significantly different in our series (*p* = 0.04), it is unlikely to be clinically relevant. Moreover, our accuracy outcomes appear comparable with previous HTO PSI series [16, 20, 21].

The accuracy evaluation (ΔMPTA, ΔmTFA and ΔMPTS) was conducted in 3D by merging the preoperative and postoperative proximal tibia model with retainment of preoperative and planned bony landmarks and axes. The methodology for accuracy measurement can hereby be considered more precise and reliable compared to other studies describing ΔMPTA accuracy results in 2D [24] or 2D versus 3D [17]. Nevertheless, a CT-scan of the knee and ankle was required in order to apply this accuracy methodology.

In the study, pain levels rapidly decreased after surgery evidenced by a significant reduction of 1.6 points at two weeks (*p* = 0.010) and 3.4 points at four weeks (*p* < 0.001). The relative immobilization period and use of pain medication immediately after surgery can partially be held responsible for low pain levels, however at four weeks, 80% was able to walk with only one crunch or none while 60% felt comfortable driving a car. After four weeks (NRS 2.7 ± 1.9), pain levels did not significantly decrease further up to one year (NRS 1.7 ± 1.9). The observation of early pain relief within the first four weeks supports previous research regarding the use of structural bone graft impaction allowing ‘weight-bearing as tolerated’ from day one [12, 25, 26]. At three months, the general KOOS outcome increased by 19.2 points which is at least comparable to most prospective HTO series publishing on short-term clinical outcomes [12, 25, 27]. The activity level improved slowly and was significantly better at one year after surgery (UCLA 7.6 ± 2.2) (*p* = 0.002).

CT-scan, 3D planning and kit preparation logistics demanded a time-interval of minimum two weeks before surgery. For kit manufacturing and medical-grade 3D printing, study hospitals collaborated with an external company (OCEANZ®, Ede, Netherlands) which occupied the majority of the preoperative timeframe. In two cases, guide transport to the hospital was compromised due to Covid-19 border restrictions but none of the surgeries had to be postponed. Total cost for guide manufacturing and transport included 180 euro/case. The 3D kit was used in combination with three different locking plate systems based on surgeons’ preference (Powerpeek (Arthrex®), Tomofix (Synthes®) and Königsee (Königsee Implantate®). This supports the accessibility of using this 3D kit in combination with multiple off-the-shelf locking plate systems. Complication rate was compliant with the reported adverse events after MOWHTO within the first year [28, 29]. Five stable lateral hinge fractures (17%), one postoperative hematoma (3%), one deep infection (3%) and one delayed union (3%) were observed. Only local implant irritation for which removal was performed appeared to be more frequent (17%) than generally reported (4.8%) [28].

The authors acknowledge certain limitations to the study. A rather small sample size (*n* = 30) was described with no conventional control group, which moderately tempers the impact of study outcomes. However, considering the first-time use of this 3D bone graft customization kit, low sample size can be defended under the heading of ‘pilot study’. Compared to the authors’ previous impaction graft study in which an accuracy outcome of 52% was reached within [-2°; + 2°], the current study showed accuracy outcomes as high as 90% [12]. 3D and 2D measurements and analysis were performed by a single observer which could have made data prone to repetitive measuring errors. Nevertheless, Victor et al. showed high reproducibility of positioning knee landmarks in the same 3D software used for this study [13]. Moreover, accuracy outcomes were evaluated as the difference in planned and postoperative alignment by merging both proximal tibia 3D models and reusing bony landmarks and axes, which makes concerns about potential preoperative bony landmark mal-positioning insignificant. Response rate to clinical questionnaires was > 90%, except at one year (73%). Missing data were processed by the linear-mixed model repeated measures approach (LMM). Finally, six subjects (20%) had a concomitant index knee procedure which could only have prolonged rehabilitation.

## Conclusion

The study suggests that 3D printed instrumentation to personalize structural bone allograft is a viable alternative method in MOWHTO that has the benefit of optimizing surgical accuracy (1.1° ± 0.7 absolute ΔMPTA) while providing early and consistent pain relief after surgery.

## Data Availability

The datasets used and/or analyzed during the current study are available from the corresponding author on reasonable request.
